# Regulatory Pathways in Growth Plate Chondrocytes that Are Impacted by Matrix Vesicle microRNA Identified by Targeted RISC Pulldown and Sequencing of the Resulting Transcriptome

**DOI:** 10.1007/s00223-023-01179-9

**Published:** 2024-02-05

**Authors:** Niels C. Asmussen, David J. Cohen, Barbara D. Boyan, Zvi Schwartz

**Affiliations:** 1 School of Integrative Life Sciences, Virginia Commonwealth University, Richmond, VA USA; 2 College of Engineering, Virginia Commonwealth University, 601 W. Main Street, Richmond, VA 23284 USA; 3 Wallace H. Coulter Department of Biomedical Engineering, Georgia Institute of Technology, Atlanta, GA USA; 4 Department of Periodontics, University of Texas Health Science Center at San Antonio, San Antonio, TX USA

**Keywords:** RISC, microRNA, mRNA, Growth plate, Matrix vesicle, Extracellular vesicles

## Abstract

During endochondral bone formation, growth plate chondrocytes are differentially regulated by various factors and hormones. As the cellular phenotype changes, the composition of the extracellular matrix is altered, including the production and composition of matrix vesicles (MV) and their cargo of microRNA. The regulatory functions of these MV microRNA in the growth plate are still largely unknown. To address this question, we undertook a targeted bioinformatics approach. A subset of five MV microRNA was selected for analysis based on their specific enrichment in these extracellular vesicles compared to the parent cells (miR-1-3p, miR-22-3p, miR-30c-5p, miR-122-5p, and miR-133a-3p). Synthetic biotinylated versions of the microRNA were produced using locked nucleic acid (LNA) and were transfected into rat growth plate chondrocytes. The resulting LNA to mRNA complexes were pulled down and sequenced, and the transcriptomic data were used to run pathway analysis pipelines. Bone and musculoskeletal pathways were discovered to be regulated by the specific microRNA, notably those associated with transforming growth factor beta (TGFβ) and Wnt pathways, cell differentiation and proliferation, and regulation of vesicles and calcium transport. These results can help with understanding the maturation of the growth plate and the regulatory role of microRNA in MV.

**Supplementary Information:**

The online version contains supplementary material available at 10.1007/s00223-023-01179-9.

## Introduction

The majority of mammalian skeletal growth occurs through the process of endochondral bone formation. In fetuses, mesenchymal stromal cells proliferate, condense, and form a cartilage template populated by chondrocytes that differentiate, mature, and trigger mineralization of the extracellular matrix (ECM), which is subsequently replaced by bone [1, 2]. During postnatal bone growth, chondrocytes in the reserve zone (resting zone cartilage, RC) form into columns and undergo proliferation. Following proliferation, cells enter the growth zone (prehypertrophic and upper hypertrophic cell zones, GC), where the chondrocytes undergo hypertrophy and mineralize the ECM [3]. The composition of ECM in RC cartilage is dominated by collagen type 2 and proteoglycan aggregates containing sulfated glycosaminoglycans, whereas in GC cartilage, the proteoglycan aggregates are degraded as the chondrocytes hypertrophy and collagen type X is synthesized [4–6]. Throughout this process chondrocytes produce a specific subgroup of extracellular vesicles called matrix vesicles (MV) and release them into their associated ECM, where they are anchored via integrins [7–9].

MVs were initially discovered as focal points of tissue mineralization with hydroxyapatite crystals forming on the inner leaflet of the phospholipid membrane, and eventually extending into the ECM [10]. Subsequent research showed that MVs have a unique composition and cargo dependent on the parent cell’s stage of maturation. MVs appear to bud off laterally from chondrocytes although the composition of their lipid membrane is distinct from that of the parent cell [11]. The cargo carried by MVs includes minerals and enzymes vital to the mineralization process but also contains factors that modulate chondrocyte function, enzymes that facilitate ECM turnover and factor activation, and microRNAs for chondrocyte regulation [12–14].

MicroRNA are short strands of RNA that are involved in the regulation of protein production within cells. Once in the cytoplasm they are loaded into the RNA Induced Silencing Complex (RISC) as ~ 22 base long single strands of RNA [15]. Depending on the complementarity of an ~ 8 base long seed region within the microRNA binding to cytoplasmic mRNA, the mRNA is either sequestered or degraded by the RISC [16, 17]. MicroRNA are typically not limited to one specific mRNA and as a result can have a wide array of effects depending on the cell’s current transcriptome [17]. This enables microRNA to have varying regulatory effects based on cell type and stage of maturation. However, this also makes a purely bioinformatic approach to predicting the mRNA target and the phenotypic effect increasingly difficult.

MV microRNAs have been demonstrated to be a population distinct from the parent cell and to not only vary based on chondrocyte maturation level but fall under the regulatory control of 1alpha,25 dihydroxy vitamin D3 [1α,25(OH)_2_D_3_] [18–20]. These microRNAs are capable of regulating the phenotype of both RC and GC chondrocytes, indicating that target pathways for the microRNA are active within the chondrocytes. It remains unclear how these microRNA find their way into chondrocytes (or possibly osteoclasts and osteoblasts involved in calcified cartilage resorption and bone formation during endochondral ossification). However, their selective export from chondrocytes into MVs with some microRNA being found almost exclusively in the cell or MV populations, their protection from RNase in MVs even following 1α,25(OH)_2_D_3_ treatment, and the increase in MVs adhering to chondrocytes treated with 1α,25(OH)_2_D_3_ all point to intentional export of the microRNAs for the purpose of downstream regulation [20].

Some of the selectively exported microRNA have known or predicted target mRNAs and pathways within chondrocytes and other cells of the musculoskeletal tissues. However comprehensive mRNA target lists for the MV microRNA remain incomplete and a large portion of the microRNA have no known or predicted targets within musculoskeletal tissues. With the high likelihood of these microRNA performing regulatory functions in growth plate chondrocytes and the potential for implementing these as treatments for growth plate disorders and possibly for use treating articular cartilage, it is important to gain a more complete understanding as to their direct regulatory role. What chondrocyte mRNA are being impacted by specific microRNA? This study used an in vitro approach to answer this question using microRNA mimic transfection, RISC pulldown, and a tabletop long read sequencer followed by bioinformatic analysis of the resulting mRNA reads and determining the pathways that are likely impacted.

## Methods

### Chondrocyte Cultures

Chondrocytes were isolated from male 100 to 125 g Sprague Dawley rats as has been previously detailed by Boyan et al*. *[3, 21]. Animal procedures connected with this work were approved by the Institutional Animal Care and Use Committee at Virginia Commonwealth University. In brief, rats were killed by CO_2_ asphyxiation followed with cervical dislocation. Sharp dissection was used to remove the rib cages and trim excess tissue. Ribs were placed in Dulbecco’s Modified Eagle’s Medium (DMEM) (Life Technologies, Carlsbad, CA) containing 1 g/L glucose, 150 U/mL penicillin and 150 μg/mL streptomycin and kept on ice. Under a dissection microscope all tissue was cut from around the ribs. The bone, GC cartilage, and RC cartilage were clearly visible under the microscope and the cartilage was carefully cut into slices using a scalpel. One or two transition slices between the RC cartilage and GC cartilage and between the GC cartilage and bone were discarded and the remaining GC slices were incubated (37˚C and 5% CO_2_) overnight in DMEM containing 1 g/L glucose, 50 U/mL penicillin, and 50 µg/mL streptomycin, plus 10% fetal bovine serum (FBS). The following day the slices were washed twice with Hank’s Balanced Salt Solution containing 50 U/mL penicillin and 50 μg/mL streptomycin then incubated in 0.25% trypsin–EDTA (Gibco, Gaithersburg, MD) for 1 h, washed once as before and incubated (37˚C and 5% CO_2_) in 0.2% collagenase type II (Worthington Biochemical, Lakewood, NJ) on a shaker for 3 h to digest the ECM. The resulting cell suspension was passed through a 40 μm nylon mesh strainer; FBS was added to 10% and the cells were pelleted by centrifugation (500 × *g*, for 10 min) followed by resuspension with DMEM Full Media (DMEM FM; DMEM with 10% FBS, 1 g/L glucose, 50 U/mL penicillin, 50 µg/mL streptomycin, and 50 µg/mL ascorbic acid). Cells were counted and then plated at a density of 20,000 cells/cm^2^. Cells were incubated (37°C and 5% CO_2_) and media were changed 24 h after plating and then every 48 h. Fourth passage cells were used for all experiments; GC chondrocytes at this passage retain their GC phenotype and provide sufficient cells for MV isolation from the ECM.

### microRNA Mimic Selection

**Fig. 1:**
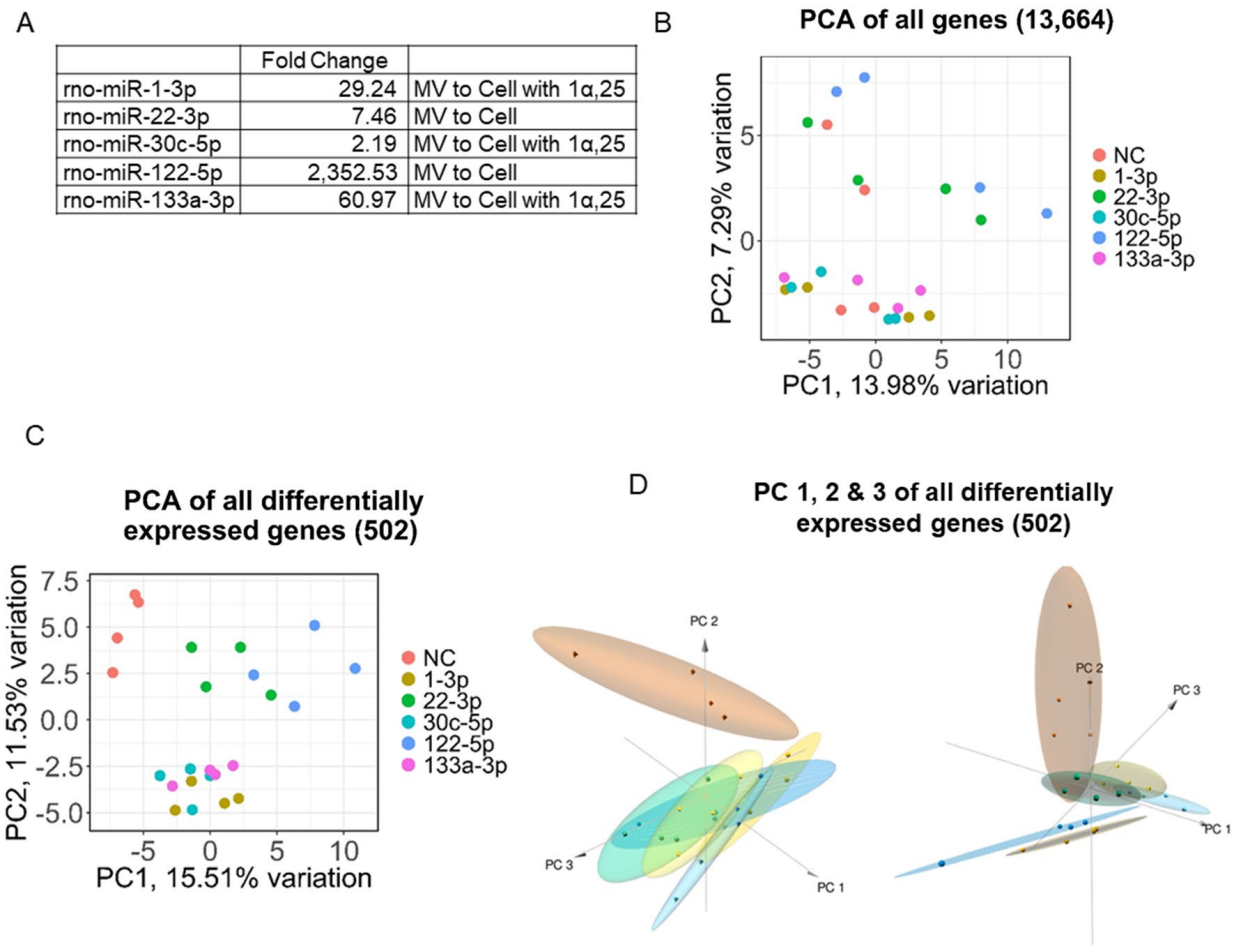
MicroRNA expression in matrix vesicles (MV) and cells and mRNA clustering after LNA based pulldown. **A** Fold changes in microRNA expression between MV and Cells. **B** 2D principal component analysis (PCA) of all 13,664 sequenced genes with PC1 on x-axis and PC2 on y-axis. **C** 2D PCA of the 502 differentially expressed genes with PC1 on x-axis and PC2 on y-axis. **D** 3D PCA of the 502 differentially expressed genes adding PC3 on the z-axis. Two different angles are displayed demonstrating separation between the six groups

We selected five microRNA that were highly exported into MVs from chondrocytes based on previous experiments [14, 19]. These microRNA exhibited fold changes ranging from 2.19 to 2,352.53 when comparing the GC microRNA populations between the cell isolation and the MVs that they produced (Fig. 1A). Three of the microRNAs were upregulated in the MVs following treatment of the chondrocytes with 1α,25(OH)_2_D_3_ and two of the microRNAs were highly enriched in the MV without treatment. Triple stranded locked nucleic acid (LNA) mimics of these microRNA biotinylated at the 3′ end were purchased from Qiagen (Hilden, Germany). In addition, mirVana™ miRNA mimics were purchased from (Thermo Fisher, Waltham, MA).

### LNA Transfection

In order to determine the optimal transfection concentration, we transfected fourth passage GC cells at 70% confluence with the LNA scrambled negative control (NC) (14.5 nM) or with different amounts of the LNA microRNA mimics 22-3p and 122-5p and measured DNA content, as described below. The scrambled NC and microRNA mimics 22-3p and 122-5p solutions were diluted 1.5X four times before combining with lipofectamine solution (final concentrations of 32.6, 21.8, 14.5, 9.7, and 6.5 nM). Cells were treated with transfection solution for 24 h before changing media to DMEM FM for another 48 h of incubation before DNA isolation and quantification. We also transfected with mirVana™ NC and microRNA mimics 22-3p and 122-5p at 14.5 nM, which we had determined in a previous study (purchased from Thermo Fisher, Waltham, MA) [14]. This was necessary as we were switching systems to a triple stranded LNA mimic and wanted to validate similar phenotypic responses in our cells that correspond to previous work.

Based on these results, fourth passage GC chondrocytes were grown to 70% confluence, media were aspirated and replaced with DMEM 1X with 10% FBS. LNA mimics were diluted 100-fold to 666.7 nM in DMEM and combined 1:1 with a solution of lipofectamine RNAiMAX transfection reagent (Invitrogen, Carlsbad, CA) that had been diluted 26-fold in DMEM. This solution of mimics and lipofectamine sat at room temperature for 20 min. Cells were treated with transfection solution for final LNA concentration of 32.6 nM and incubated in transfection media for 48 h before RISC pulldown and RNA isolation.

### DNA Quantification

DNA quantity was assayed and compared with previous results to determine optimal transfection concentrations of the new type of microRNA mimics. The media were aspirated and the cell layers washed twice with 1X PBS. 100 µL of 0.05% Triton- × 100 in H_2_O was added per well and the plate moved to −80 °C for storage. Samples were thawed on ice and sonicated (40 amps, 10 s per well). The QuantiFluor dsDNA system (Promega, Madison, WI) was used for quantification following standard protocol with samples diluted 1:10 and read on a plate reader (Synergy H1 Hybrid Reader, BioTek, Winooski, VT) with excitation of 485 nm and emission of 538 nm.

### RISC PullDown and RNA Isolation

RISC and RNA isolation were performed using a protocol based on Dash et al. [22] 30 µL per sample of streptavidin coated magnetic beads (Pierce, Waltham, MA) were washed the day before cell harvest (3 washes with 100 µL of 10 mM Tris–Cl pH 7.5, 0.5 mM EDTA, 1 M NaCl solution and 3 washes with 100 µL solution of 0.1 M NaOH, 0.05 M NaCl), resuspended (100 µL solution of 0.5 M NaCl), and blocked (200 µL of 1 μg/μL BSA, 2 μg/μL yeast tRNA solution) overnight in 4 ˚C. Cells were removed from flasks with trypsin, pelleted (1500 × g for 5 min at 4  C), washed with sterile 1X Dulbecco’s phosphate buffered saline (DPBS) (Cytiva, Marlborough, MA), pelleted again as before, and resuspended in 600 µL lysis buffer [150 mM NaCl, 25 mM Tris–HCl pH 7.5, 5 mM DL-dithiothreitol (DTT) (Sigma-Aldrich, St. Louis, MO, USA), 0.5% octylphenoxy poly(ethyleneoxy)ethanol (IGEPAL) (Sigma-Aldrich, St. Louis, MO, USA), 60 U/mL Superase, 1 × protease inhibitor cocktail] before being rapidly frozen in −80 °C and then thawed on ice. Cellular debris was pelleted (16,000 × g for 5 min at 4 °C) and the supernatant combined with ¼ volume of 5 M NaCl to generate cell lysate solution. Beads from previous day were washed three times with 150 µL of pulldown wash buffer (10 mM KCl, 1.5 mM MgCl2, 10 mM Tris–HCl pH 7.5, 5 mM DTT, 1 M NaCl, 0.5% IGEPAL, 60 U/mL Superase, 1 × protease inhibitor cocktail) and then resuspended in 300 µL of pulldown wash buffer. 300 µL of cell lysate solution was incubated with 300 µL streptavidin coated beads for one hour at room temperature. Beads were washed three times with 300 µL pulldown wash buffer and finally resuspended in 100 µL of nuclease free water on ice. 700 µL qiazol (Qiagen) was added to each tube before transferring to −80 °C. RNA was precipitated following miRNeasy micro kit (Qiagen). RNA was eluted in 30 μL of nuclease free water per sample.

### Library Preparation and RNAseq

RNA isolations were quantified with a RNA 6000 pico chip on a BioAnalyzer (Agilent, Santa Clara, CA) and library prepared according to specifications of the PCR-cDNA Barcoding kit (SQK-PCB109, Oxford Nanopore, Oxford, UK) for 12 samples and run on a Spot On Flow Cell Mk 1 R9 (Oxford Nanopore) for 72 h. High precision nucleic acid basecalling was carried out on Nanopore’s MinIT running MinKNOW (21.05.24) and guppy (5.0.16) to determine the sequence.

### Bioinformatic Analysis

Fastq files that were considered to have passed basecalling were transferred to VCU’s high performance research computing core facility where reads were aligned using minimap2 (2.21-r1071) to Ensemble’s *Rattus norvegicus* transcriptome (Rnor_6.0) and quantified with salmon (v1.5.2) [23, 24]. The count data were then analyzed in R (4.1.0), differential expression determined using DESeq2 (1.32.0), PCA plots built with pca3d (0.10.2) and PCAtools (2.4.0), Venn diagrams made with VennDiagram (1.6.20), heatmap with pheatmap (1.0.12), and volcano plots and histograms with ggplot2 (3.3.5) [25–31]. UTRdb was used to download the 3′ untranslated regions (UTRs) of the differentially expressed genes [32]. Specific microRNA sequences were downloaded from mirBase.org [33]. The RNAhybrid tool was used to determine the microRNA to mRNA 3′UTR minimum binding energies [34]. Pathway analysis was carried out using the PANTHER classification system (16.0) webtool [35].

### Statistical Analysis

DNA quantity is presented as mean ± standard error of the mean for six samples for each group. An ANOVA with Tukey HSD post-hoc test was used to examine differences between the groups. Significance was determined by a p > 0.05.

## Results

### microRNA 22 & 122 Transfection Concentration

LNA mimics had similar but not identical effects on DNA content to the mirVana mimics (Supplemental Fig. 1). While cells transfected with LNA NC were comparable to the non-treated control cells, chondrocytes transfected with mirVana NC had slightly elevated DNA content. At 32.6 nM, LNA 122-5p mimic caused a comparable increase in DNA to mirVana mimic 122-5p. In contrast, the DNA content of cells transfected with 32.6 nM LNA 22-3p was slightly greater than seen in cells transfected with mirVana mimic 22-3p.

### Sample Clustering

13,664 genes were returned from the alignment of the sequencing reads between all 24 samples. A PCA plot of these reads resulted in minimal clustering with just over 20% of variation represented between PC 1 & 2 (Fig. 1B). Setting a cutoff for differential expression at a p-value ≤ 0.05 and an absolute log twofold change > 1 and comparing each treatment microRNA with the NC group resulted in 502 total genes between all five comparisons. Re-examining only the 502 differentially expressed genes between each target microRNA and NC resulted in three distinct clusters with NC alone in one cluster, microRNAs 22-3p and 122-5p having minimal overlap in a second cluster and microRNAs 1-3p, 30c-5p, and 133a-3p grouping together in a third cluster (Fig. 1C). A scree plot of the differentially expressed genes shows that roughly 35% of variation is explained by PCs 1–3 (Supplemental Fig. 2A). This can be visualized in a 3d PCA plot, which shows distinct clusters forming when viewed with PCs 1–3 (Fig. 1D).

### Differential Expression

**Fig. 2:**
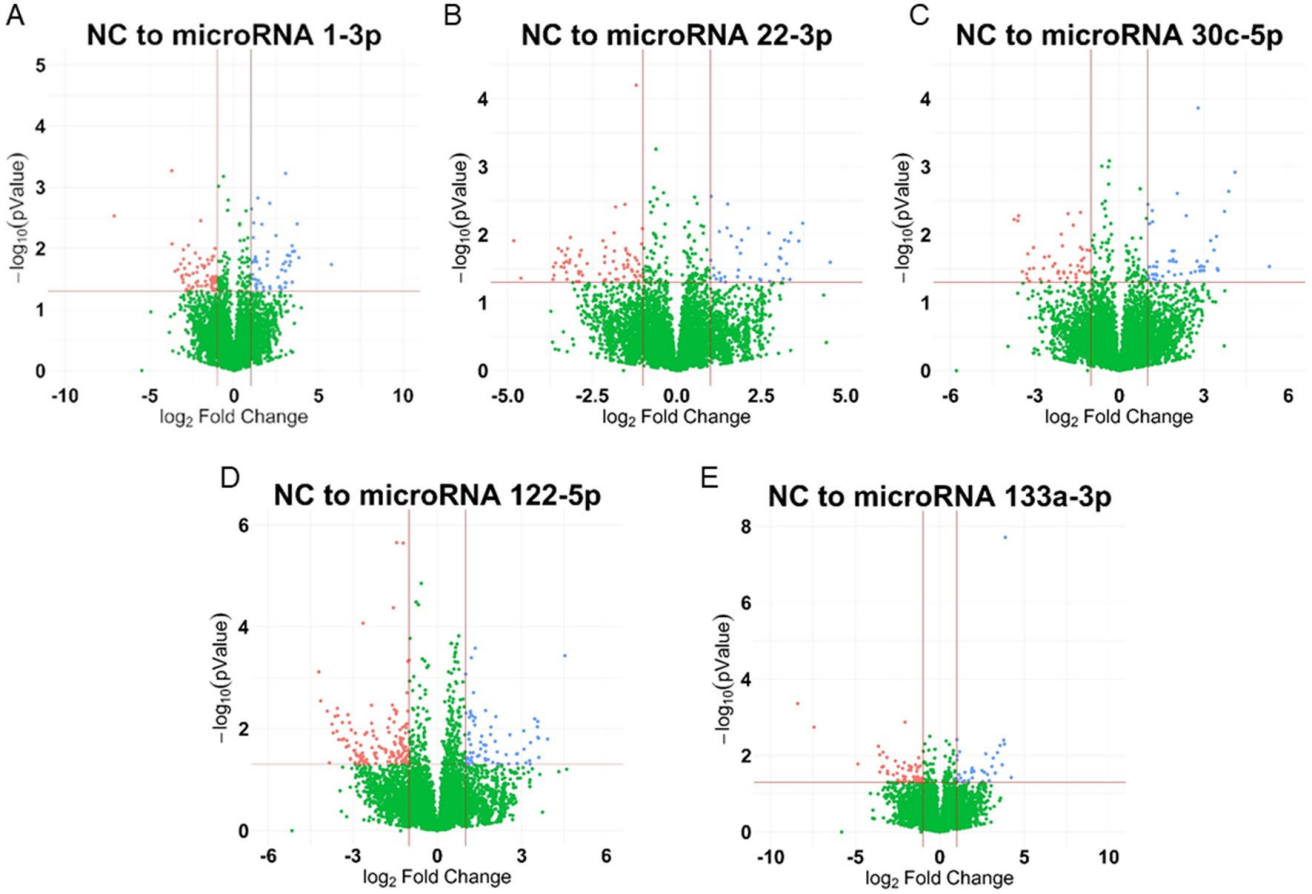
Volcano plots of all the sequenced genes for each microRNA compared to the scrambled negative control (NC). Fold change values have been log2 transformed and the p-values were -log10 transformed

**Fig. 3:**
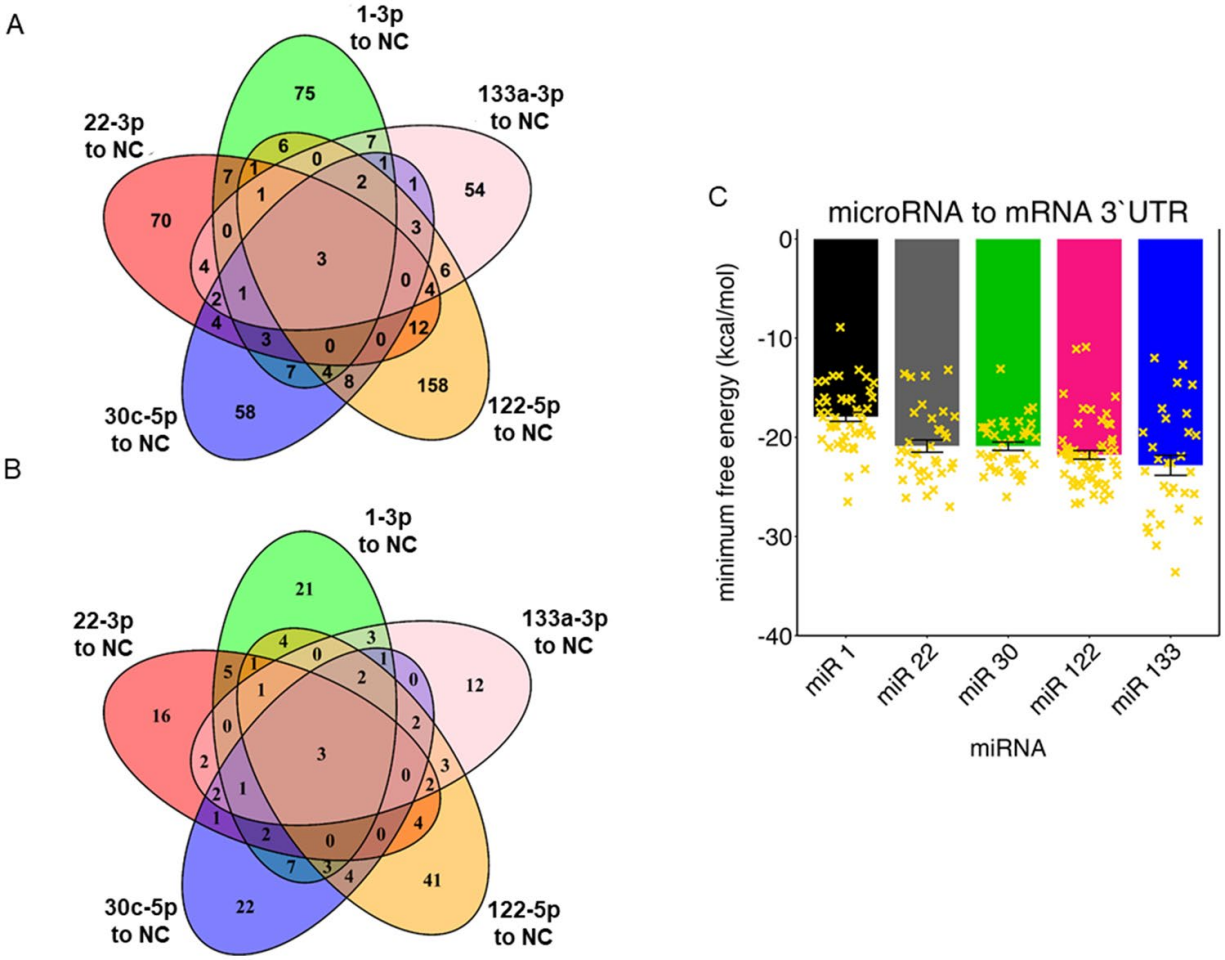
**A** Overlap of all the differentially expressed genes between each of the five microRNA and the scrambled negative control (NC). **B** Overlap of only the differentially expressed genes that were enriched when comparing each of the five microRNA to NC. **C** Predicted minimum free energy values for each specific microRNA and the differentially expressed and enriched genes

MicroRNA groups (1-3p, 22-3p, 30c-5p, 122-5p, and 133a-3p) are compared against NC using DESeq2 with a cut off for differential expression of absolute log twofold change > 1 and a p-value ≤ 0.05. Results of all five comparisons were visualized in volcano plots with a horizontal line at –log_10_(0.05) and vertical lines at log_2_(0.5) and log_2_(2) (Fig. 2A–E, Supplemental Fig. 2B). All five comparisons had differentially up and down regulated genes compared to NC. Examining all 502 differentially expressed genes (increased or decreased) between all five comparisons for overlap, 415 of the genes were only differentially expressed within one comparison and 62 were shared by two comparisons, with the remaining 25 shared by three or more (Fig. 3A). Of the 502 total differentially expressed genes, 165 of them were increased in the LNA microRNA sample compared to NC, indicating active binding to the respective LNA mimic. 112 (67.9%) of these were uniquely increased within one microRNA and 33 (20.0%) were shared between two with the remaining 20 (13.1%) shared by three or more microRNA (Fig. 3B and Supplemental Table 1).

### microRNA to mRNA Binding Energy

The RNAhybrid command line tool was used to predict the minimum free energy (MFE) of the specific microRNA with the differentially expressed upregulated mRNA’s 3′ UTR when comparing the microRNA in question with NC. The average MFE for all of the microRNA to target mRNA combinations was −20.8 kcal/mol (Fig. 3C). The MFE prediction was based on the most advantageous binding location estimated within the provided 3′ UTR target sequence.

### Pathways

Results from the PANTHER classification system were scanned for musculoskeletal related terms (Supplemental Table 2) and then filtered for a p-value ≤ 0.05. The results indicated that all of the microRNA are capable of regulating the expression of proteins connected to pathways involved in bone and skeletal development, cell division, and cell differentiation. MicroRNA 1-3p is focused on transforming growth factor beta and Wnt pathways along with cell differentiation and proliferation. MicroRNA 22-3p, 30c-5p, and 122-5p have many different functions. They are the three microRNA from our group that primarily regulate the loading, development, transport and release of vesicles (matrix vesicles). MicroRNA 133a-3p regulates vesicles and calcium transport (Table 1).

**Table 1:**
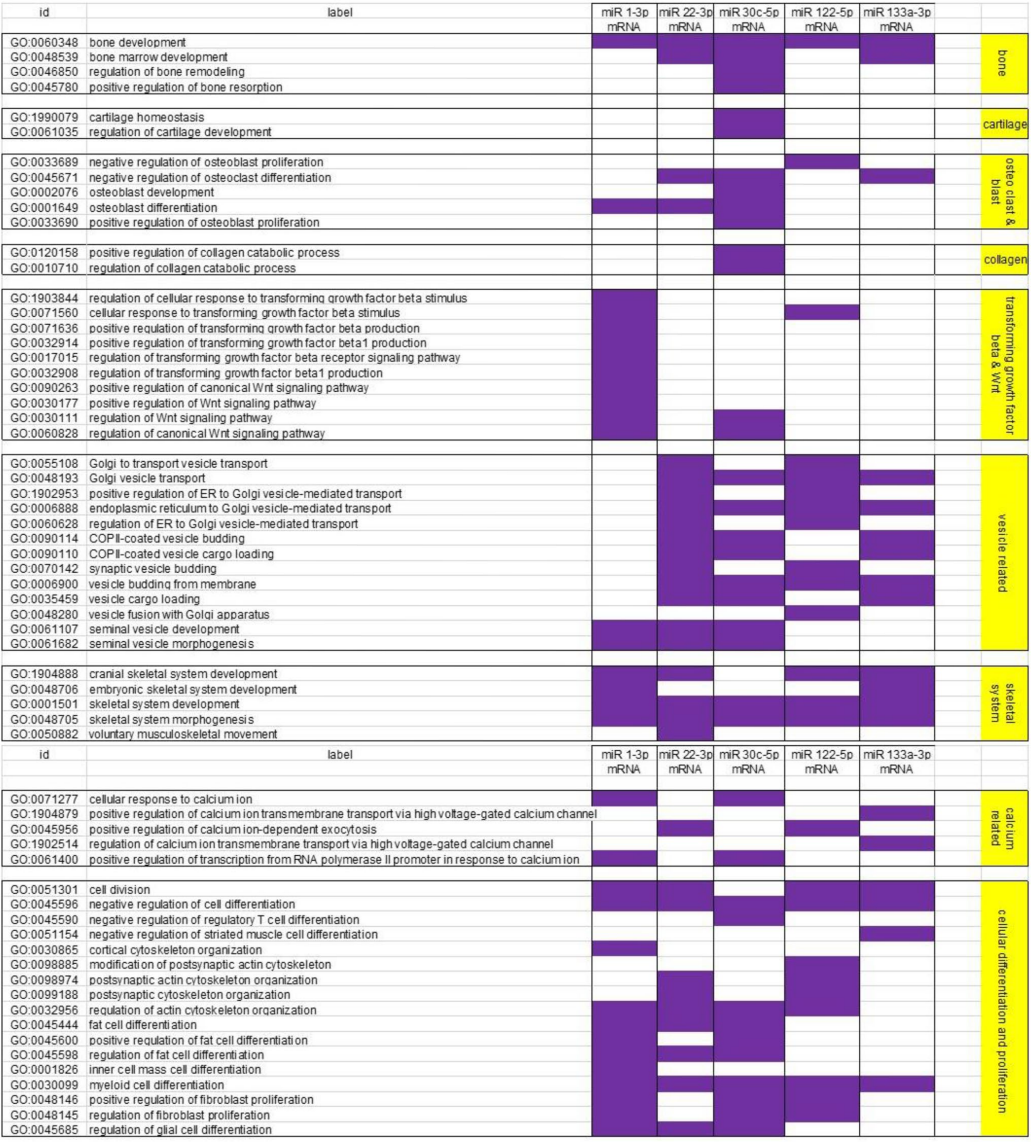
PANTHER results for the enriched differentially expressed genes after filtering for musculoskeletal related terms (Supplemental Table 2). Purple indicates active involvement in that pathway with a p-value ≤ 0.05 for genes enriched by the corresponding microRNA

## Discussion

This study aimed to establish an approach to gain a more complete understanding of what impact specific MV microRNA may be having in GC chondrocytes during growth plate maturation. The specific microRNAs in question were studied previously in other tissues and have lists of predicted targets within the rat genome. However, these putative target lists only provide a glimpse of what may be happening within the cells as the cell type and active transcriptome determine what effect the microRNA are able to elicit. Therefore, we sought to find an approach that would allow us to directly examine what mRNA are being regulated by a given set of microRNAs.

This approach required a switch in the type of mimics being used for transfection, so we began by analyzing the response of the GC cells to the biotin tagged LNA mimics of two previously well studied microRNA (22-3p and 122-5p) [14, 19]. We selected DNA production as a robust response indicator of microRNA transfection based on previously published work in our lab [14]. The highest concentration of LNA mimic was selected for the transfections as it produced the most consistent phenotypic response with our previous research. Moreover, because the objective was to pulldown active RISC, the potential side effects of disproportionally occupying the RISC with the mimic and thereby blocking endogenous microRNA from being loaded and regulating the cells was not a large concern in this study.

Initial examination of the sequencing results produced 13,664 genes between the six groups. Many of these genes had very low copy counts in the raw data and when selecting for the 502 genes found to be differentially expressed between NC and the select microRNA we were able to get more distinct clustering of the six groups. The magnetic beads were blocked with yeast tRNA to reduce off target RNA binding during the pulldown steps and the beads went through numerous wash steps as part of the RNA isolation procedure. However, this may only produce incomplete reduction of noise in the actual pulldown procedure.

The differentially expressed genes had both increased and decreased sub-populations in comparison to NC and when all five comparisons were examined for overlap the majority of genes were found to be unique within their group. We focused on the mRNA found to be increased in the specific microRNA compared to NC group as we were looking for the mRNA pulled down as part of the RISC. Searching through the prediction databases, most of the mRNA coming up as increased in our specific microRNA were not found as likely targets. To try and further evaluate the likelihood of these being accurate mRNA targets we examined the MFE between each microRNA and the 3′ UTR of their differentially expressed up-regulated mRNA targets. The MFE prediction was based on the most advantageous binding location estimated within the provided 3′ UTR target sequence. The average MFE for all of our microRNA to mRNA bindings was −20.8 kcal/mol, which is slightly higher than the −25 to −30 kcal/mol often used by prediction algorithms [36, 37]. While the binding energies were somewhat higher than what computational tools would employ as a cutoff, we don’t believe that these values are outside the range of biologically likely interactions. These data show that the microRNA binding to the RISC complex was specific compared to the NC binding, and validates the expression results of the present study.

We used the increased differentially expressed mRNA to run a pathway analysis, scanned the results for musculoskeletal related terms and filtered by p-value ≤ 0.05. The results can be organized into categories and we see some distinct patterns emerge with microRNA 30c-5p involved in bone, cartilage, osteoblast and osteoclast development, and collagen catabolism. MicroRNA 1-3p is involved in multiple aspects of the transforming growth factor beta (TGFβ1) and Wnt pathways. MicroRNA 22-3p and to a lesser extent microRNAs 30c-5p and 122-5p are involved with vesicle related pathways. MicroRNA 133a-3p is involved in various aspects of skeletal development and all the microRNA except for 122-5p are involved to some degree in calcium pathways and all five microRNAs have an assortment of cellular differentiation and proliferation pathways that they impact.

The p-value cutoff for the pathway analysis results is likely on the conservative end as we were investigating the mRNA binding partners of a specific microRNA and the regulatory effects of individual microRNA may not shut down an entire pathway but may instead exert significant modulations on a specific pathway. This should also be taken into consideration in light of an individual microRNA being part of a larger population of microRNAs that may be delivered by MVs as regulators of the cells. Combining the mRNA impacted by numerous microRNA into one pathway search may well produce a more accurate examination of the regulatory potential of a specific group of microRNAs.

The present study demonstrates the ability to pulldown and sequence the mRNA being targeted by specific microRNA that were highly exported by chondrocytes into MVs and had the potential to regulate the musculoskeletal system though how the microRNAs are able to enact this regulatory role from the MVs is still unclear. We were able to use the mRNA sequence results to analyze impacted pathways. Conducted for a larger group of microRNAs that form a regulatory population, the process could provide higher confidence estimates of impacted pathways and a better understanding of the role played by MVs and their specific microRNA. For example, microRNA-1-3p is packaged in MVs in response to 1α,25(OH)_2_D_3_ [20] along with matrix metalloproteinase 3 [38, 39], which activates latent TGFβ1 in the ECM [40, 41]. In the present study, we show that microRNA-1-3p produced by GC chondrocytes and exported in MVs is able to bind to mRNA within the TGFβ1 pathways and likely modulate them. This suggests that MV components may play multiple roles in the growth plate, regulating availability of matrix bound factors like TGFβ1 and ensuring that appropriate cells are competent to respond.

Although this procedure is far more involved than a simple database search, the results it produces are specific to the tissue under investigation and provide insight into the observed phenotypic responses observed in cell culture. MicroRNA 122-5p was previously found to increase chondrocyte proliferation while microRNA 22-3p had no effect [14], and this was reflected in our related pathways with microRNA 122-5p being associated with two proliferation pathways and microRNA 22-3p not being associated with any.

While we carried out transfection and pulldown using individual microRNA, it is conceivable that a set of tagged LNA could be transfected and pulled down together to increase the throughput of this approach. As more attention is being given to tissue specific microRNA-based therapies, it is important to have a more robust understanding of what mRNA and pathways are being impacted by a given set of microRNAs. This is important to understand how the desired regulatory effect is being achieved as well as to increase the likelihood of early detection of potential undesired side effects.

## Conclusion

This study focused on microRNA that have been demonstrated to be exported by chondrocytes in MVs and are presumed to be acting as regulatory messengers. By transfecting mimics of these microRNA into chondrocytes we were able to isolate a population of mRNA that were being regulated within this tissue’s active transcriptome. Examining the pathways that the sequenced mRNAs were involved in yielded a high number of cartilage, bone, vesicle, and other skeletal tissue related processes. Given that, GC MVs are involved in mediating events at the transition of calcified cartilage to bone during endochondral ossification.

This study demonstrates the effectiveness of this approach in evaluating the mRNA targeted by specific microRNAs within the active transcriptome of select cells leading to a more thorough understanding of the microRNA’s role within tissues.

### Supplementary Information

Below is the link to the electronic supplementary material.

Supplementary file1 (PPTX 932 KB)

## Abbreviations

1α,25(OH)_2_D_3_: 1α,25-Dihydroxyvitamin D3
24R,25(OH)_2_D_3_: 24R,25-dihydroxyvitamin D3
ECM: Extracellular Matrix
GC: Growth Zone Cartilage
MV: Matrix Vesicles
RC: Resting Zone Cartilage
UTR: Untranslated Region
