# Supplementary material for: Regulatory Pathways in Growth Plate Chondrocytes that Are Impacted by Matrix Vesicle microRNA Identified by Targeted RISC Pulldown and Sequencing of the Resulting Transcriptome
Source: Calcif Tissue Int. 2024 Feb 5;114(4):409–18. doi: 10.1007/s00223-023-01179-9 (PMC10957581; doi:10.1007/s00223-023-01179-9)
Supplement: Supplementary file 1 — Supplementary file1 (PPTX 932 KB) [file 223_2023_1179_MOESM1_ESM.pptx]

## Slide 1
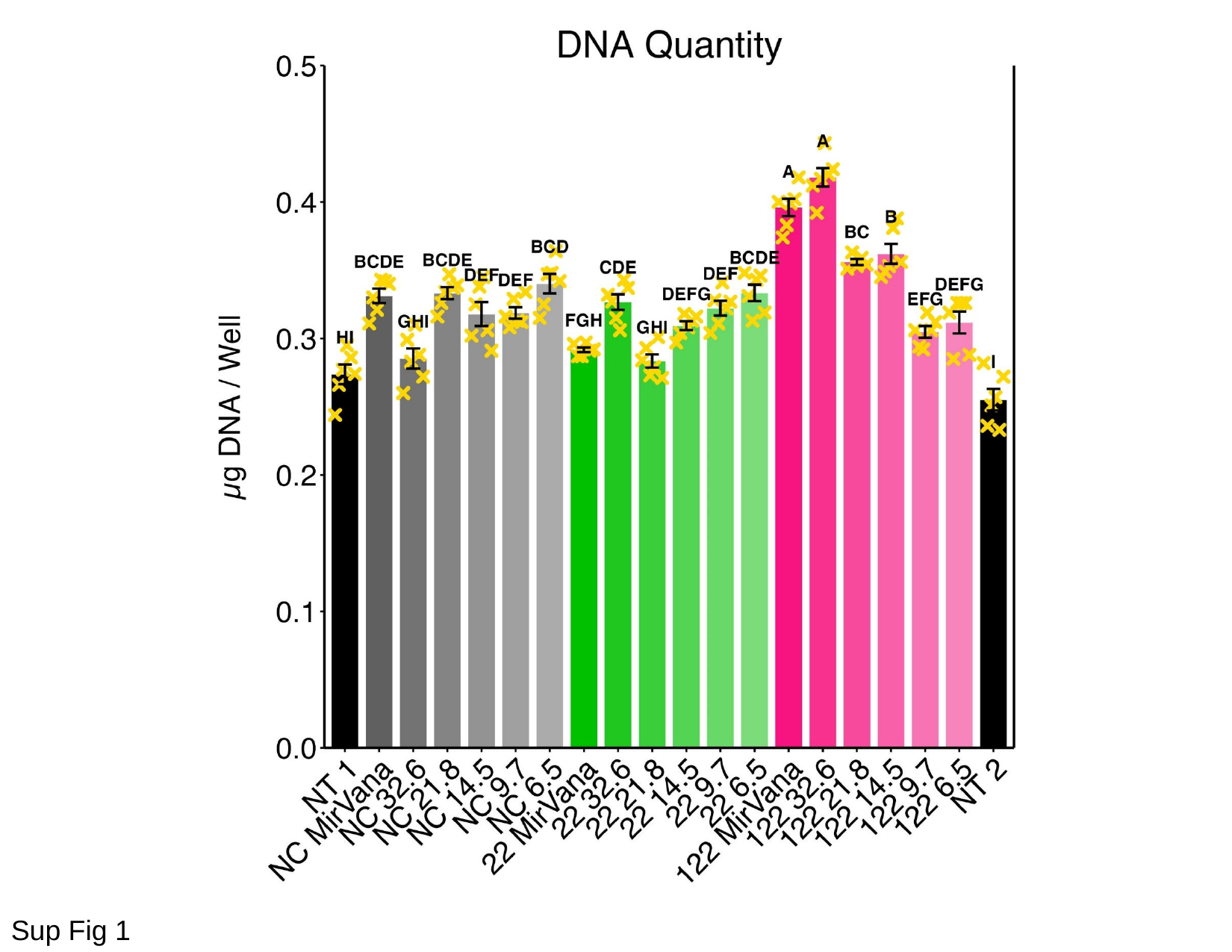

Sup Fig 1

## Slide 2
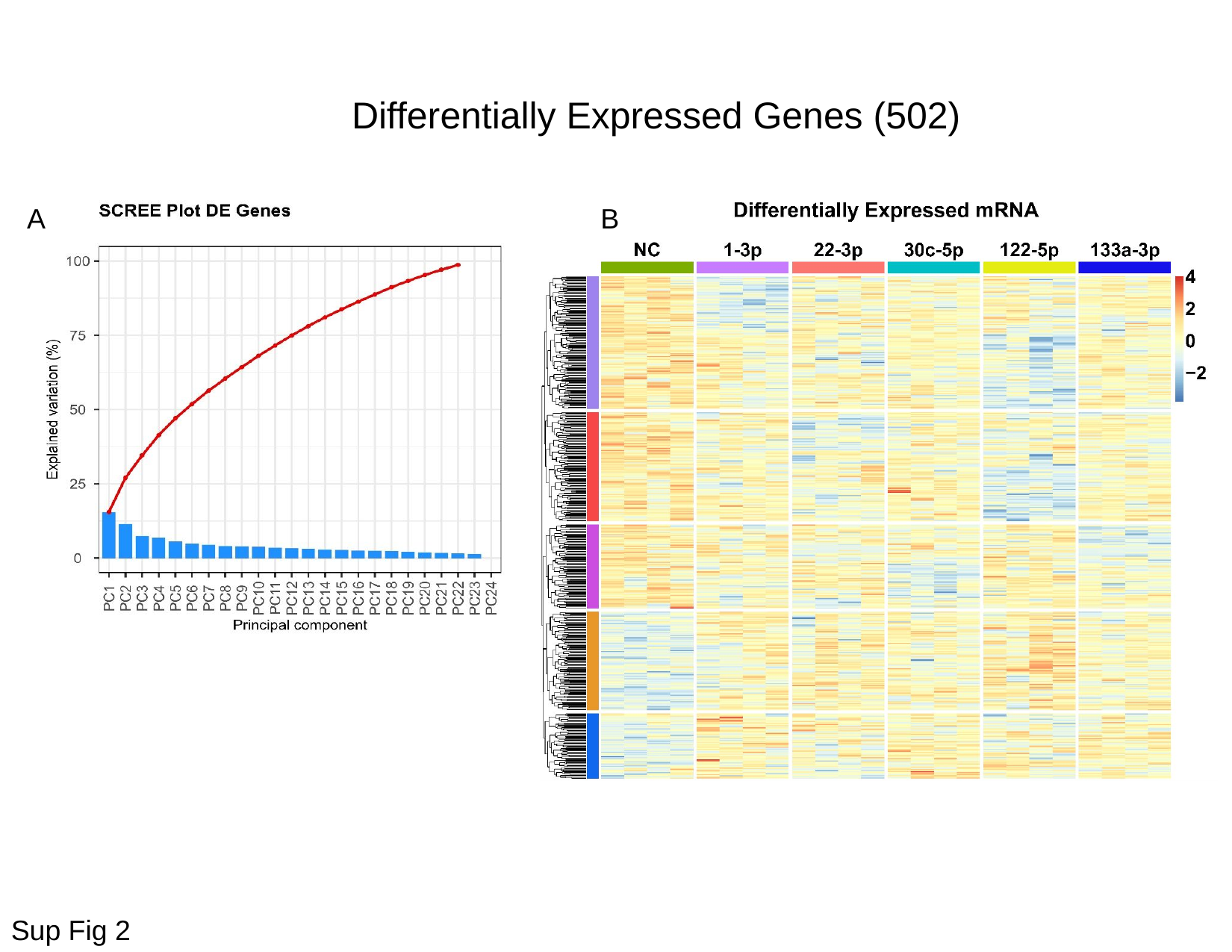

Differentially Expressed Genes (502)
A
B
Sup Fig 2

## Slide 3
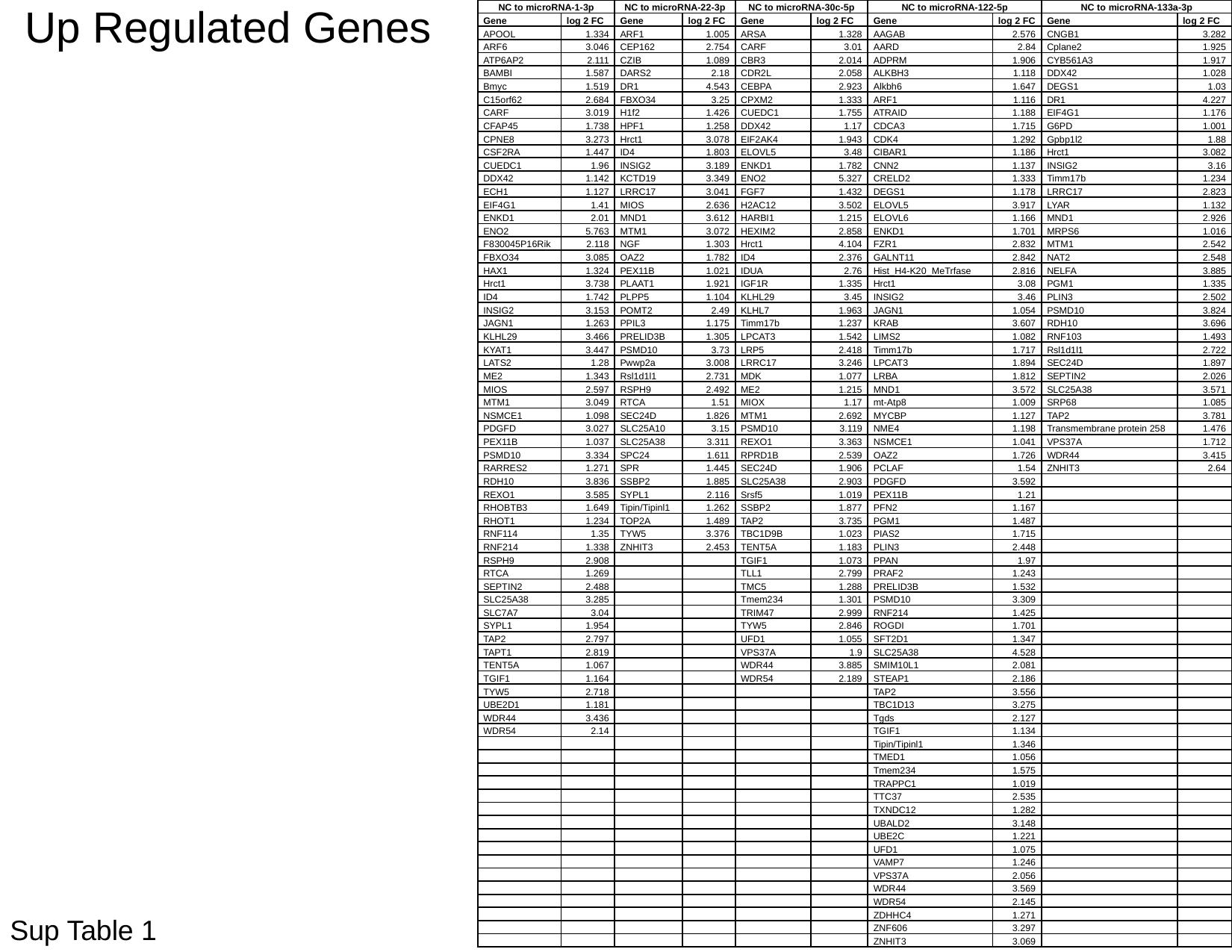

# Up Regulated Genes
| NC to microRNA-1-3p | | NC to microRNA-22-3p | | NC to microRNA-30c-5p | | NC to microRNA-122-5p | | NC to microRNA-133a-3p | |
| --- | --- | --- | --- | --- | --- | --- | --- | --- | --- |
| Gene | log 2 FC | Gene | log 2 FC | Gene | log 2 FC | Gene | log 2 FC | Gene | log 2 FC |
| APOOL | 1.334 | ARF1 | 1.005 | ARSA | 1.328 | AAGAB | 2.576 | CNGB1 | 3.282 |
| ARF6 | 3.046 | CEP162 | 2.754 | CARF | 3.01 | AARD | 2.84 | Cplane2 | 1.925 |
| ATP6AP2 | 2.111 | CZIB | 1.089 | CBR3 | 2.014 | ADPRM | 1.906 | CYB561A3 | 1.917 |
| BAMBI | 1.587 | DARS2 | 2.18 | CDR2L | 2.058 | ALKBH3 | 1.118 | DDX42 | 1.028 |
| Bmyc | 1.519 | DR1 | 4.543 | CEBPA | 2.923 | Alkbh6 | 1.647 | DEGS1 | 1.03 |
| C15orf62 | 2.684 | FBXO34 | 3.25 | CPXM2 | 1.333 | ARF1 | 1.116 | DR1 | 4.227 |
| CARF | 3.019 | H1f2 | 1.426 | CUEDC1 | 1.755 | ATRAID | 1.188 | EIF4G1 | 1.176 |
| CFAP45 | 1.738 | HPF1 | 1.258 | DDX42 | 1.17 | CDCA3 | 1.715 | G6PD | 1.001 |
| CPNE8 | 3.273 | Hrct1 | 3.078 | EIF2AK4 | 1.943 | CDK4 | 1.292 | Gpbp1l2 | 1.88 |
| CSF2RA | 1.447 | ID4 | 1.803 | ELOVL5 | 3.48 | CIBAR1 | 1.186 | Hrct1 | 3.082 |
| CUEDC1 | 1.96 | INSIG2 | 3.189 | ENKD1 | 1.782 | CNN2 | 1.137 | INSIG2 | 3.16 |
| DDX42 | 1.142 | KCTD19 | 3.349 | ENO2 | 5.327 | CRELD2 | 1.333 | Timm17b | 1.234 |
| ECH1 | 1.127 | LRRC17 | 3.041 | FGF7 | 1.432 | DEGS1 | 1.178 | LRRC17 | 2.823 |
| EIF4G1 | 1.41 | MIOS | 2.636 | H2AC12 | 3.502 | ELOVL5 | 3.917 | LYAR | 1.132 |
| ENKD1 | 2.01 | MND1 | 3.612 | HARBI1 | 1.215 | ELOVL6 | 1.166 | MND1 | 2.926 |
| ENO2 | 5.763 | MTM1 | 3.072 | HEXIM2 | 2.858 | ENKD1 | 1.701 | MRPS6 | 1.016 |
| F830045P16Rik | 2.118 | NGF | 1.303 | Hrct1 | 4.104 | FZR1 | 2.832 | MTM1 | 2.542 |
| FBXO34 | 3.085 | OAZ2 | 1.782 | ID4 | 2.376 | GALNT11 | 2.842 | NAT2 | 2.548 |
| HAX1 | 1.324 | PEX11B | 1.021 | IDUA | 2.76 | Hist\_H4-K20\_MeTrfase | 2.816 | NELFA | 3.885 |
| Hrct1 | 3.738 | PLAAT1 | 1.921 | IGF1R | 1.335 | Hrct1 | 3.08 | PGM1 | 1.335 |
| ID4 | 1.742 | PLPP5 | 1.104 | KLHL29 | 3.45 | INSIG2 | 3.46 | PLIN3 | 2.502 |
| INSIG2 | 3.153 | POMT2 | 2.49 | KLHL7 | 1.963 | JAGN1 | 1.054 | PSMD10 | 3.824 |
| JAGN1 | 1.263 | PPIL3 | 1.175 | Timm17b | 1.237 | KRAB | 3.607 | RDH10 | 3.696 |
| KLHL29 | 3.466 | PRELID3B | 1.305 | LPCAT3 | 1.542 | LIMS2 | 1.082 | RNF103 | 1.493 |
| KYAT1 | 3.447 | PSMD10 | 3.73 | LRP5 | 2.418 | Timm17b | 1.717 | Rsl1d1l1 | 2.722 |
| LATS2 | 1.28 | Pwwp2a | 3.008 | LRRC17 | 3.246 | LPCAT3 | 1.894 | SEC24D | 1.897 |
| ME2 | 1.343 | Rsl1d1l1 | 2.731 | MDK | 1.077 | LRBA | 1.812 | SEPTIN2 | 2.026 |
| MIOS | 2.597 | RSPH9 | 2.492 | ME2 | 1.215 | MND1 | 3.572 | SLC25A38 | 3.571 |
| MTM1 | 3.049 | RTCA | 1.51 | MIOX | 1.17 | mt-Atp8 | 1.009 | SRP68 | 1.085 |
| NSMCE1 | 1.098 | SEC24D | 1.826 | MTM1 | 2.692 | MYCBP | 1.127 | TAP2 | 3.781 |
| PDGFD | 3.027 | SLC25A10 | 3.15 | PSMD10 | 3.119 | NME4 | 1.198 | Transmembrane protein 258 | 1.476 |
| PEX11B | 1.037 | SLC25A38 | 3.311 | REXO1 | 3.363 | NSMCE1 | 1.041 | VPS37A | 1.712 |
| PSMD10 | 3.334 | SPC24 | 1.611 | RPRD1B | 2.539 | OAZ2 | 1.726 | WDR44 | 3.415 |
| RARRES2 | 1.271 | SPR | 1.445 | SEC24D | 1.906 | PCLAF | 1.54 | ZNHIT3 | 2.64 |
| RDH10 | 3.836 | SSBP2 | 1.885 | SLC25A38 | 2.903 | PDGFD | 3.592 | | |
| REXO1 | 3.585 | SYPL1 | 2.116 | Srsf5 | 1.019 | PEX11B | 1.21 | | |
| RHOBTB3 | 1.649 | Tipin/Tipinl1 | 1.262 | SSBP2 | 1.877 | PFN2 | 1.167 | | |
| RHOT1 | 1.234 | TOP2A | 1.489 | TAP2 | 3.735 | PGM1 | 1.487 | | |
| RNF114 | 1.35 | TYW5 | 3.376 | TBC1D9B | 1.023 | PIAS2 | 1.715 | | |
| RNF214 | 1.338 | ZNHIT3 | 2.453 | TENT5A | 1.183 | PLIN3 | 2.448 | | |
| RSPH9 | 2.908 | | | TGIF1 | 1.073 | PPAN | 1.97 | | |
| RTCA | 1.269 | | | TLL1 | 2.799 | PRAF2 | 1.243 | | |
| SEPTIN2 | 2.488 | | | TMC5 | 1.288 | PRELID3B | 1.532 | | |
| SLC25A38 | 3.285 | | | Tmem234 | 1.301 | PSMD10 | 3.309 | | |
| SLC7A7 | 3.04 | | | TRIM47 | 2.999 | RNF214 | 1.425 | | |
| SYPL1 | 1.954 | | | TYW5 | 2.846 | ROGDI | 1.701 | | |
| TAP2 | 2.797 | | | UFD1 | 1.055 | SFT2D1 | 1.347 | | |
| TAPT1 | 2.819 | | | VPS37A | 1.9 | SLC25A38 | 4.528 | | |
| TENT5A | 1.067 | | | WDR44 | 3.885 | SMIM10L1 | 2.081 | | |
| TGIF1 | 1.164 | | | WDR54 | 2.189 | STEAP1 | 2.186 | | |
| TYW5 | 2.718 | | | | | TAP2 | 3.556 | | |
| UBE2D1 | 1.181 | | | | | TBC1D13 | 3.275 | | |
| WDR44 | 3.436 | | | | | Tgds | 2.127 | | |
| WDR54 | 2.14 | | | | | TGIF1 | 1.134 | | |
| | | | | | | Tipin/Tipinl1 | 1.346 | | |
| | | | | | | TMED1 | 1.056 | | |
| | | | | | | Tmem234 | 1.575 | | |
| | | | | | | TRAPPC1 | 1.019 | | |
| | | | | | | TTC37 | 2.535 | | |
| | | | | | | TXNDC12 | 1.282 | | |
| | | | | | | UBALD2 | 3.148 | | |
| | | | | | | UBE2C | 1.221 | | |
| | | | | | | UFD1 | 1.075 | | |
| | | | | | | VAMP7 | 1.246 | | |
| | | | | | | VPS37A | 2.056 | | |
| | | | | | | WDR44 | 3.569 | | |
| | | | | | | WDR54 | 2.145 | | |
| | | | | | | ZDHHC4 | 1.271 | | |
| | | | | | | ZNF606 | 3.297 | | |
| | | | | | | ZNHIT3 | 3.069 | | |
Sup Table 1

## Slide 4
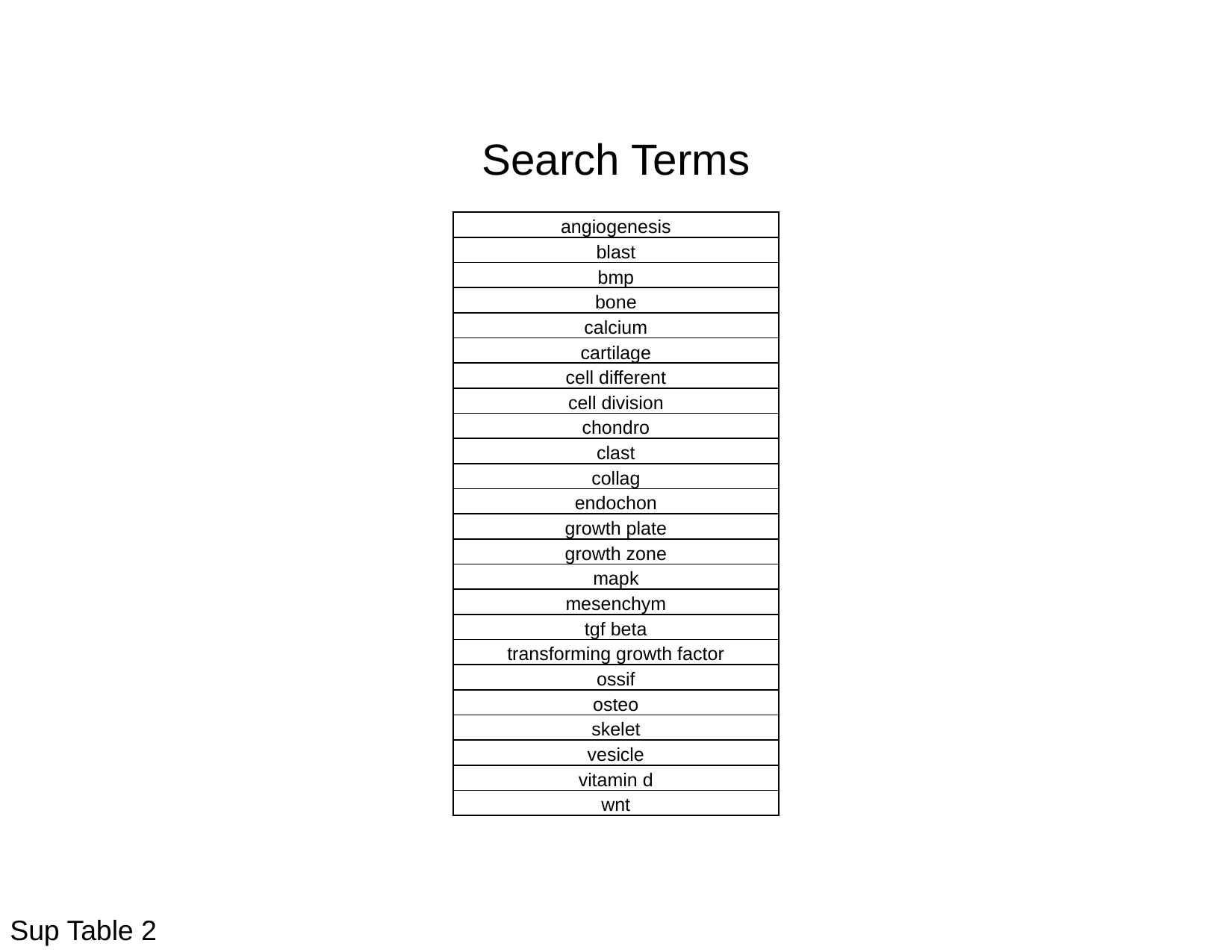

# Search Terms
| angiogenesis |
| --- |
| blast |
| bmp |
| bone |
| calcium |
| cartilage |
| cell different |
| cell division |
| chondro |
| clast |
| collag |
| endochon |
| growth plate |
| growth zone |
| mapk |
| mesenchym |
| tgf beta |
| transforming growth factor |
| ossif |
| osteo |
| skelet |
| vesicle |
| vitamin d |
| wnt |
Sup Table 2
